# Ouabain Suppresses the Migratory Behavior of Lung Cancer Cells

**DOI:** 10.1371/journal.pone.0068623

**Published:** 2013-07-10

**Authors:** Varisa Pongrakhananon, Preedakorn Chunhacha, Pithi Chanvorachote

**Affiliations:** Department of Pharmacology and Physiology, Faculty of Pharmaceutical Sciences and Cell-based Drug and Health Product Development Research Unit, Chulalongkorn University, Bangkok, Thailand; Cincinnati Children's Hospital Medical Center, United States of America

## Abstract

The migratory capability of cancer cells is one of the most important hallmarks reflecting metastatic potential. Ouabain, an endogenous cardiac glycoside produced by the adrenal gland, has been previously reported to have anti-tumor activities; however, its role in the regulation of cancer cell migration remains unknown. The present study has revealed that treatment with ouabain at physiological concentrations is able to inhibit the migratory activities of human lung cancer H292 cells. The negative effects of ouabain were found to be mediated through the suppression of migration regulatory proteins, such as focal adhesion kinase (FAK), ATP-dependent tyrosine kinase (Akt), and cell division cycle 42 (Cdc42). We found that the observed actions of ouabain were mediated via a reactive oxygen species (ROS)-dependent mechanism because the addition of ROS scavengers (N-acetylcysteine and glutathione) could reverse the effect of ouabain on cell migration. Furthermore, ouabain was shown to inhibit the spheroidal tumor growth and decrease the cancer cell adhesion to endothelial cells. However, the compound had no significant effect on anoikis of the cells. Together, these findings shed light on the understanding of cancer cell biology by exploring the novel function of this endogenous human substance.

## Introduction

Understanding the molecular basis of cancer cells in response to biologically derived substances is considered an essential aid to discovering novel molecular targets for drug therapies. Substantial evidence has indicated that ouabain, a sodium/potassium pump inhibitor, is present in human plasma and tissues in the range of 0.002-0.77 nM [1]–[3]. In addition, ouabain was found to be up-regulated in several pathologies including cardiac failure [1], [4], renal failure [5] and hypertension [3], [6]. Recently, we have provided evidence indicating that ouabain sensitizes tumor necrosis factor-related apoptosis-inducing ligand (TRAIL)-mediated lung cancer cell apoptosis, and this finding suggests that ouabain may affect cancer cell biology [7].

In lung cancer, metastasis has become the most interesting area of research because the high death rate of this disease is associated with cancer metastasis [8]. During cell spreading, the cancer cells must have the ability to migrate from their original sites into the nearby circulatory system. Increased kinase activity of focal adhesion kinase (FAK), a key signaling pathway controlling cell motility, potentiates tumorigenesis and metastasis [9]. Such alterations were also found during the acquisition process of metastatic cancer cells [10], [11]. FAK controls signal transduction from integrin-enriched focal adhesion sites of the cell-extracellular matrix interaction [12]. Regarding the regulation of cancer cell migration, FAK phosphorylation at Tyr-397 and the recruitment of Src family kinases are important processes that initiate migration [12]–[13]. Additionally, the activated status of several migratory regulators such as ATP-dependent tyrosine kinase (Akt) and cell division cycle 42 (Cdc42) [14], [15] are critical for the process of cell movement. Several studies have indicated that activation of Akt enhances the capability of cancer cells to migrate and invade [15], [16]. Akt that is localized at the edge of moving cells interacts with actin-binding proteins and induces actin remodeling and the formation of membrane protrusions, facilitating cell motility [15]. This concept was confirmed by a study showing that the down-regulation of Akt using an antisense technique causes a dramatic inhibition of cancer cell invasion *in vitro* [17] and *in vivo* [18]. Additionally, the interaction of FAK and ERK1/2 has been shown to control cell motility [19]–[20]. Recently, the Rho family of small guanosine triphosphatases (GTPases), especially Cdc42, has been shown to play an essential role in actin reorganization and filopodia formation. The expression level of Cdc42 was found to be up-regulated in many cancers including lung cancer [21]–[22], and Cdc42 induction was shown to enhance cancer migration [23].

Thus far, the role of endogenous levels of ouabain in the regulation of cancer cell motility has been largely unknown, and such an understanding could lead to novel anti-cancer therapies. Therefore, we aimed to investigate the possible impact of this biological substance on cancer cell migration and invasion in non-small cell lung cancer cells. For the first time, we show that endogenous levels of ouabain suppress tumor cell motility and are associated with decreased activation of FAK and Akt and expression of Cdc42. Our study suggests the novel hypothesis that this physiological substance has a negative regulatory role in the process of cancer cell metastasis.

## Materials and Methods

### Cells and Reagents

The non-small cell lung cancer (NSCLC) cell lines H292 and H460 were obtained from the American Type Culture Collection (Manassas, VA). The cells were cultured in RPMI 1640 supplemented with 5% fetal bovine serum (FBS), 2 mM L-glutamine and 100 units/mL penicillin/streptomycin. The cells were incubated in a 5% CO_2_ environment at 37°C. Ouabain, dichlorofluorescein diacetate (DCFH_2_-DA), poly 2-hydroxyethylmethacrylate (poly-HEMA), 3-(4,5-dimethyl-thiazol-2-yl)-2,5-diphenyl tetrazolium bromide (MTT) and phalloidin tetramethylrhodamine B isothiocyanate were obtained from Sigma Chemical, Inc. (St. Louis, MO). Propidium iodide (PI) and Hoechst 33342 were obtained from Molecular Probes, Inc. (Eugene, OR). Antibodies for pan-Akt, p473-Akt, FAK, p397-FAK, Cdc42, caveolin-1 and β-actin, as well as peroxidase-conjugated secondary antibodies were obtained from Cell Signaling Technology, Inc. (Danvers, MA).

### Cell Viability and Apoptosis Assays

Cell viability was evaluated using the MTT assay. After the indicated treatments, the cells were incubated with 500 µg/ml of MTT at 37°C for 4 h. An intensity reading of the MTT product was measured at 550 nm using a microplate reader, and the percentage of viable cells was calculated in relation to control cells. Apoptosis was determined by Hoechst 33342/PI staining and DNA content analysis. The cells were washed and incubated with 10 µg/ml Hoechst 33342 and 5 µg/ml PI for 30 min. Nuclei condensation and DNA fragmentation of apoptotic cells and PI-positive necrotic cells were visualized and scored by fluorescence microscopy (Olympus IX51 with DP70). For sub G0 DNA content analysis, after the specified treatment, the cells were trypsinized, washed with phosphate-buffered saline (PBS) and fixed in 70% ethanol at 37°C for 3 h. After washing with phosphate-buffered saline, the cells were incubated in a PI solution containing 0.1% Triton-X, 1 µg/ml RNase and 1 mg/ml propidium iodide at room temperature for 30 min. The DNA content was analyzed using flow cytometry (FACSort, Becton Dickinson, Rutherford, NJ).

### ROS Detection

Intracellular ROS levels were determined by flow cytometry using DCFH-DA as a fluorescent probe. Briefly, the cells were incubated with 10 µM DCFH_2_-DA for 30 min at 37°C, after which they were washed, trypsinized, resuspended in phosphate-buffered saline, and immediately analyzed for fluorescence intensity by a microplate reader.

### Migration Assay

Migration was determined by wound healing and transwell assays. For the wound healing assay, a monolayer of cells was cultured in a 24-well plate, and a wound space was made with a 1-mm-wide tip. After rinsing with PBS, the cell monolayers were incubated with the indicated treatments and allowed to migrate for 24 h. Micrographs were taken under a phase contrast microscope (Olympus DP70, Melville, NY), and the wound spaces were measured from 10 random fields of view using Olympus DP controller software. Quantitative analysis of cell migration was performed using an average wound space from those random fields of view, and the percentage of change in the wound space was calculated using the following formula: % change = (average space at time 0 h) - (average space at time 24 h)/(average space at time 0 h) × 100. Relative cell migration was calculated by dividing the percentage change in the wound space of treated cells by that of the control cells in each experiment. For the transwell assay, the cells were seeded at a density of 5×10^4^ cells/well onto the upper chamber of a transwell (8 µm pore size) in a 24-well plate in serum-free medium and incubated with various concentrations of ouabain. RPMI medium containing 10% FBS was added to the lower chamber. Following the incubation, the non-migrated cells in the upper chamber were removed by cotton-swab wiping, and the cells that migrated to the underside of the membrane were stained with 10 µg/ml Hoechst 33342 for 10 min and visualized and scored under a fluorescence microscope (Olympus IX51 with DP70).

### Invasion Assay

An invasion assay was performed using a 24-well transwell unit with polycarbonate (PVDF) filters (8 µm pore size). The membrane was coated with 0.5% matrigel on the upper surface of the chamber overnight at 37°C in a humidified incubator. The cells were plated at a density of 2 × 10^4^ cells per well into the upper chamber of the transwell unit in serum-free medium. Medium containing 10% FBS was added to the lower chamber of the unit. After incubation with specific test agents for 24 h at 37°C, the medium in the upper chamber was aspirated, and the cells on the upper side of the membrane were removed with a cotton swab. The cells that invaded to the underside of the membrane were stained with 10 µg/ml Hoechst 33342 for 10 min, visualized and scored under a fluorescence microscope.

### Cell Morphology and Filopodia Characterization

Cell morphology was investigated by a phalloidin-rhodamine and sulforhodamine B staining assay. The cells were seeded at a density of 10^4^ cells/well onto a 96-well plate overnight. The cells were treated with various concentrations of ouabain for 24 h. The cells were then washed with PBS, fixed with 4% paraformaldehyde in PBS for 10 min at 37*°*C, permeabilized with 0.1% Triton-X100 in PBS for 4 min, and blocked with 0.2% BSA for 30 min. Then, the cells were incubated with either 1∶100 phalloidin-rhodamine in PBS or 0.4% sulforhodamine B in 1% acetic acid for 15 min, rinsed 3 times with PBS, and mounted with 50% glycerol. Cell morphology was then assessed by fluorescent imaging (Olympus IX51 with DP70). Filopodia protrusion was represented as the average number of filopodia/cell relative to untreated cells in each field.

### In vitro 3D Tumorigenesis Assay

In vitro 3D tumorigenesis was performed in a matrigel-coated 96-well plate. A plate was coated with 0.5% matrigel and left for solidification overnight at 37*°*C. The cells were suspended in culture medium containing 4% matrigel and ouabain (0–30 pM), and plated at a density of 10^3^ cells/well onto a matrigel-coated plate. Medium containing ouabain (0–30 pM) was replaced every 3 days. After 10 days, the cells were visualized and scored by image analyzer under microscope.

### Anoikis Assay

For anoikis evaluation, Tissue culture six-well plates were coated with 6 mg/ml poly(2-hydroxyethyl-methacrylate (poly-HEMA) (Sigma-Aldrich, St. Louis, MO) in 95% ethanol and incubated at 37°C for drying. H292 cells were plated in poly-HEMA-coated plates in RPMI medium containing indicated treatments for 0–24 h. After incubation with 10 µg/ml Hoechst 33342 for 30 min, nuclei condensation and DNA fragmentation of anoikis cells were visualized and scored by fluorescence microscopy (Olympus IX51 with DP70).

### Anchorage-independent Growth Assay

Anchorage-independent growth was determined by colony formation assay in soft agar. Briefly, cells from 6-well plate monolayer cultures were prepared into a single-cell suspension. Cells were suspended in RPMI containing 10% FBS and 0.33% low melting temperature agarose, and 2×10^4^ cells were plated in a 35 mm dish over a layer of solidified RPMI/10% FBS/0.6% agarose. The cells were fed every three days by adding 200 µl of RPMI/10% FBS. Colonies were stained using 10 µg/ml Hoechst 33342 for 30 min, visualized and scored by image analyzer under fluorescent microscope (Olympus IX51 with DP70).

### Monolayer Cell Adhesion Assay

HUV-EC-C were stimulated with 10 ng/ml IL-1β for 0–4 h. After indicated treatment, H292 cells (2.5 × 10^4^ cells/ml) were added onto a semi-confluent monolayer culture of HUV-EC-C, incubated for 20 min at 37°C with rotation at 120 rpm, and washed extensively to exclude nonspecific cell attachment. The number of attached cells was counted directly under a fluorescence microscope.

### Western Blotting

After specific treatments, the cells were incubated in lysis buffer containing 20 mM Tris-HCl (pH 7.5), 1% Triton X-100, 150 mM sodium chloride, 10% glycerol, 1 mM sodium orthovanadate, 50 mM sodium fluoride, 100 mM phenylmethylsulfonyl fluoride and a commercial protease inhibitor cocktail (Roche Molecular Biochemicals) for 30 min on ice. The cell lysates were collected, and the protein content was determined using the Bradford method (Bio-Rad Laboratories, Hercules, CA). Equal amounts of protein from each sample (60 µg) were denatured by heating at 95°C for 5 min with Laemmli loading buffer and subsequently loaded onto a 10% SDS-polyacrylamide gel. After separation, the proteins were transferred onto 0.45-µm nitrocellulose membranes (Bio-Rad). The transferred membranes were blocked for 1 h in 5% nonfat dry milk in TBST (25 mM Tris-HCl (pH 7.5), 125 mM NaCl, 0.05% tween 20) and incubated with the appropriate primary antibodies at 4°C overnight. The membranes were washed twice with TBST for 10 min and incubated with horseradish peroxidase-coupled isotype-specific secondary antibodies for 1 h at room temperature. The immune complexes were detected by enhancing with a chemiluminescence substrate (Supersignal West Pico; Pierce) and quantified using analyst/PC densitometry software (Bio-Rad).

### Statistical Analysis

Mean data from independent experiments were normalized to the results from cells in the control group. All of the experiments were repeated at least four times. A statistical analysis between two groups was verified by Student’s t test, and to compare to multiple groups, an analysis of variance (ANOVA) with a post-hoc test was conducted. A P-value of less than 0.05 was considered statistically significant.

## Results

### Migratory Characteristic of Non-small Cell Lung Cancer H292 Cells

**Figure 1:**
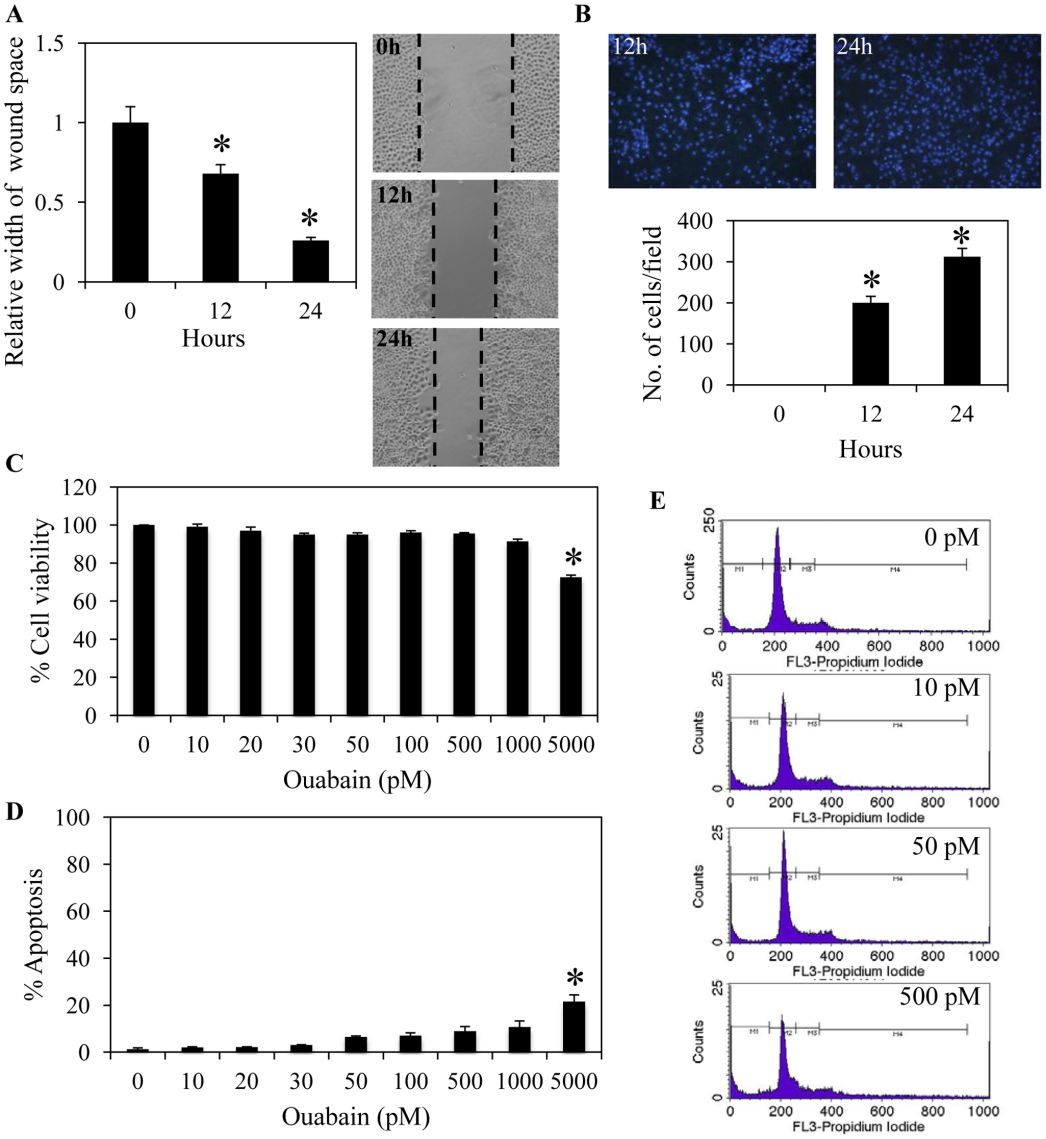
Characterization of human lung cancer H292 cell migration. A: Confluent monolayers of H292 cells were wounded using a 1-mm-wide tip and allowed to migrate for 0–24 h. The wound space was visualized under a microscope, and the relative migration levels compared with the 0 h time point were calculated. Data represent the means ± SD (n = 4). *P<0.05 vs. value at the 0 h time point. B: H292 cell migration was examined by a transwell assay for 0–24 h. Migratory cells at the basolateral side of the membrane were stained with Hoechst 33342 for 30 min and visualized under a fluorescence microscope. Data were plotted as an average number of cells in each field and represent the means ± SD (n = 4). *P<0.05 vs. value at 0 h time point. C: H292 cells were treated with various concentrations of ouabain (0–5,000 pM) for 24 h. Cell survival was determined by a 3-(4,5-dimethly-thiazol-2-yl)-2,5-diphenyl tetrazolium bromide (MTT) assay. The viability of untreated cells was represented as 100%. Data represent the means ± SD (n = 4). *P<0.05 vs. untreated control cells. D: Apoptotic cell death was evaluated using Hoechst 33342 staining. Data represent the means ± SD (n = 4). *P<0.05 vs. untreated control cells. E: Cellular apoptosis was determined by DNA content analysis using flow cytometry.

To test the effect of ouabain on lung cancer cell migration, we first characterized the migration profile of H292 cells using wound migration and transwell assays. Figs. 1A and B show that H292 cells migrated across the wound space in a time-dependent manner, and the results were consistent with those obtained from the transwell migration assay.

Next, a cytotoxicity experiment was performed to test whether biological concentrations of ouabain affect H292 cell viability. The cells were cultured in the absence or presence of ouabain (10–5000 pM), and the viability of the cells was determined using an MTT assay at 24 h. The results indicate that concentrations of ouabain at endogenous levels (2–770 pM) caused neither toxic nor proliferative effects on these lung cancer cells (Fig. 1C). While the concentrations of this substance up to 1000 nM induced no apoptosis, 5000 nM ouabain significantly reduced cell viability (Fig. 1 C) and mediated cell death detected by a Hoechst 33342 nuclear staining assay (Fig. 1D). In addition, the cells were treated with 0–500 pM ouabain for 24 h, and the cells were subjected to flow cytometry for sub G_0_ fraction determination. Fig. 1E shows that treatment of the cells with 10–500 pM ouabain caused no significant alteration of the sub G_0_ fraction in comparison to that of the untreated control cells. These results suggest that concentrations of ouabain found in human plasma and tissues do not affect the viability of H292 cells.

### Ouabain Inhibits Lung Cancer Cell Migration and Invasion

**Figure 2:**
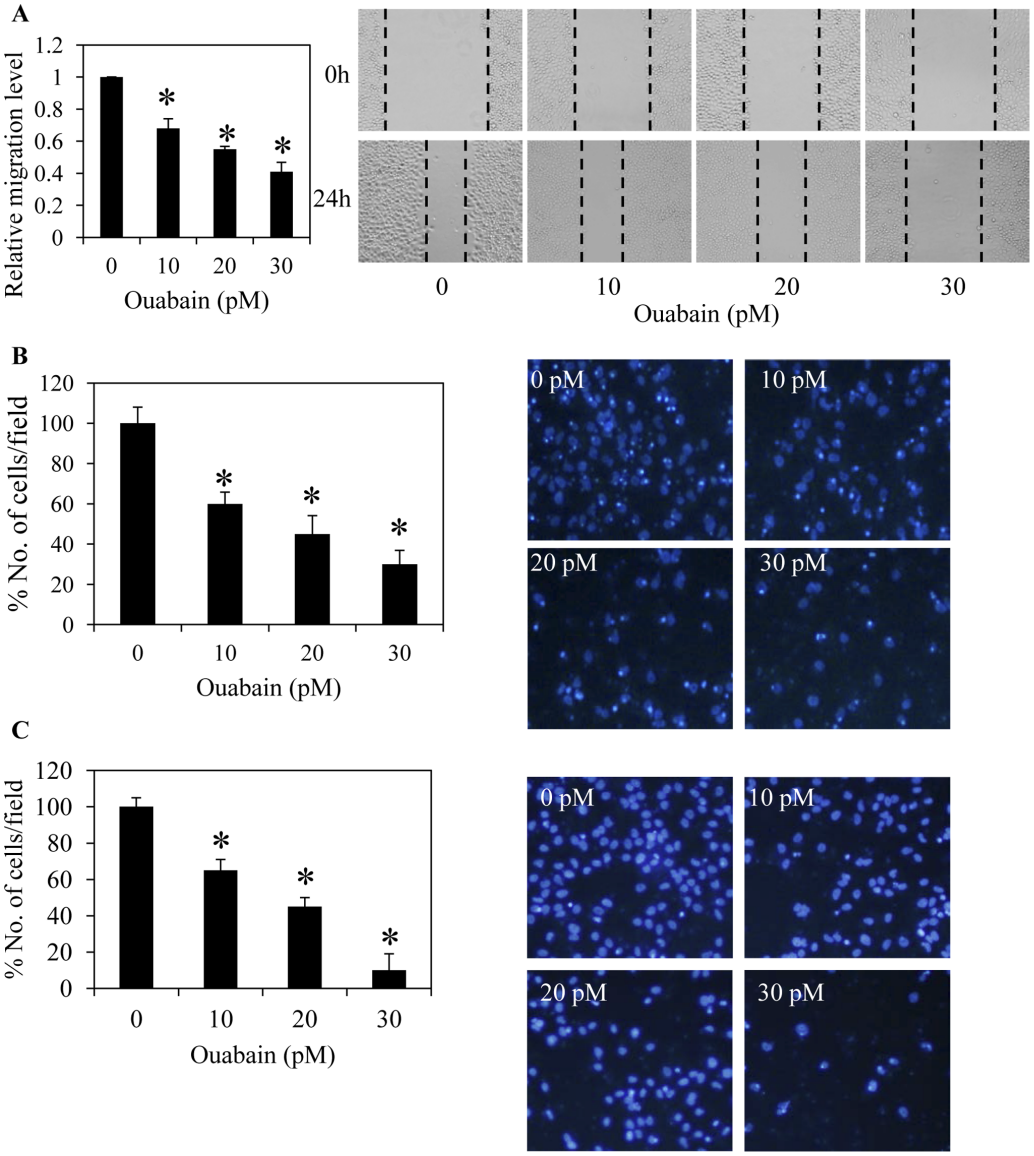
Ouabain inhibits lung cancer cell migration and invasion. A: Confluent monolayers of H292 cells were wounded using a 1-mm-wide tip and incubated with ouabain (0–30 pM) for 24 h. The wound space was analyzed and represented as the migration level relative to the change of the untreated cells. B: Migratory cells were stained with Hoechst 33342 for 30 min, visualized under a fluorescence microscope and represented as an average number of cells in each field. C: Cell invasion was evaluated using a transwell coated with matrigel as described under *Materials and Methods*. After 24 h, the cells that invaded across the membrane were visualized under a fluorescence microscope and represented as an average number of cells in each field. Data represent the means ± SD (n = 4). *P<0.05 vs. untreated control cells.

Endogenous concentrations of ouabain were further tested for their possible effect on cancer cell migration. The cells were treated with 0–30 pM ouabain and used in migration assays for 24 h. A wound migration assay showed that ouabain suppressed H292 cell migration in a dose-dependent manner compared with the untreated control cells (Fig. 2A). At the concentration of 20 pM, ouabain caused an approximate 50% reduction in the migratory activity of the cells. Additionally, the transwell migration assay provided supporting data indicating that ouabain significantly inhibited cell migration (Fig. 2B). Although the defined mechanisms and regulation of cancer cell migration and invasion are not completely known, studies have suggested that there are associated signaling pathways [24]. The effect of ouabain on the invasive potential of cancer cells was further tested using an extracellular matrix-coated transwell assay. The cells were added to the transwell and treated with the indicated concentrations of ouabain for 24 h. Fig. 2C shows that ouabain treatment substantially reduced the number of invaded H292 cells compared with untreated control cells with an approximate 50% reduction found in response to 20 pM ouabain.

### Ouabain Reduces Filopodia Formation in H292 Cells

**Figure 3:**
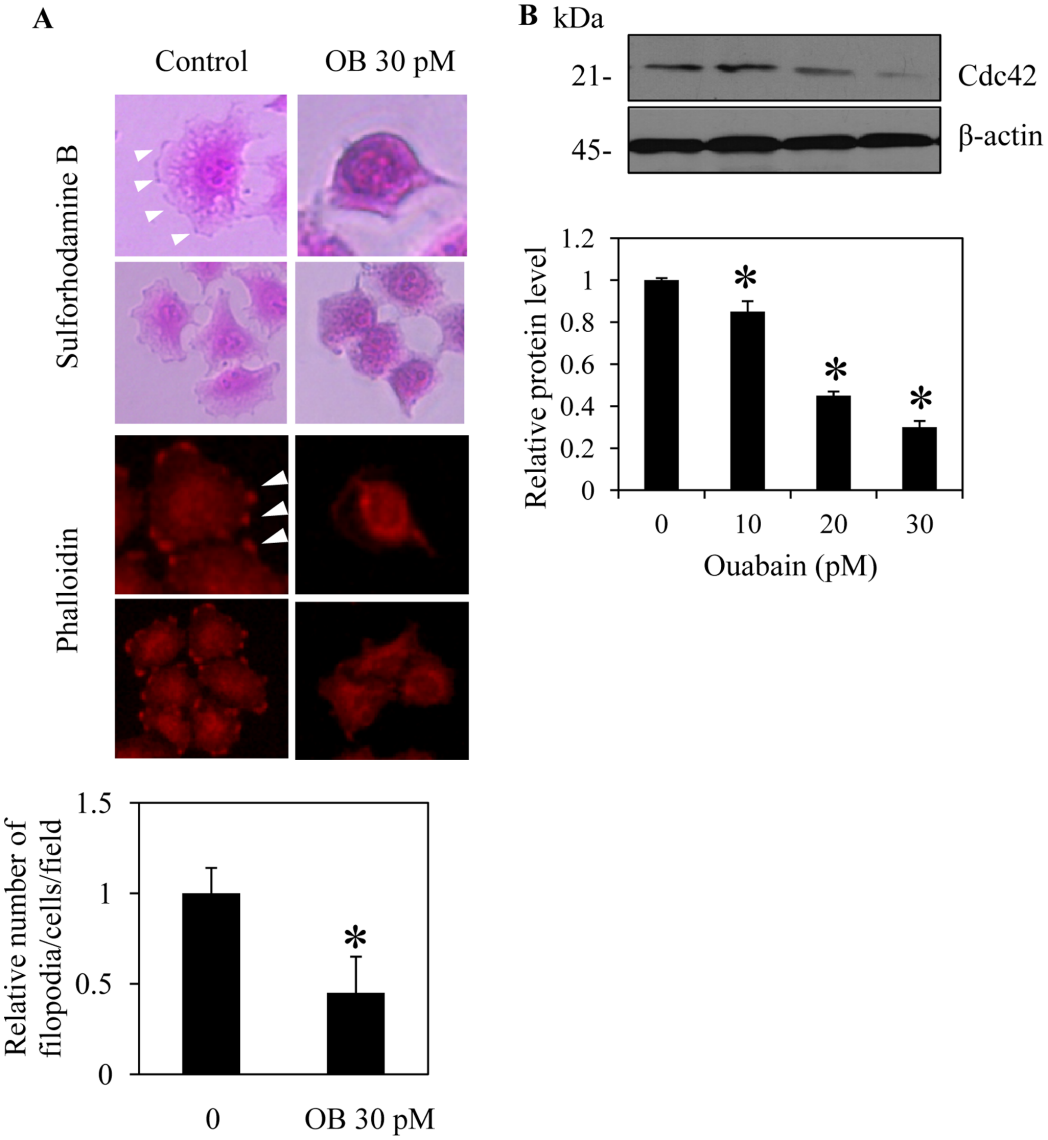
Ouabain attenuates filopodia formation. A: H292 cells were treated with 30 pM ouabain (OB) or left untreated as control cells for 24 h. The cells were stained with either sulforhodamine B or phalloidin and visualized under a fluorescence microscope at 40×. Filopodia protrusions are indicated by arrows and are represented as an average number of filopodia per cell in each field relative to untreated cells. Data represent the means ± SD (n = 4). *P<0.05 vs. untreated control cells. B: After the indicated treatment, the cells were collected and analyzed for cell division cycle 42 (Cdc42) expression by Western blotting. The blots were reprobed with β-actin to confirm equal loading. The immunoblot signals were quantified by densitometry, and mean data from independent experiments were normalized to the results. The bars are the means ± SD (n = 4). *P<0.05 vs. untreated control cells.

Plasma membrane protrusions called filopodia increase during cell movement, and the formation of filopodia is involved in cancer cell migration and metastasis [25]. Having shown that ouabain inhibited the migration and invasion of the cells, we further investigated the effect of this substance on the presence of filopodia in H292 cells. The cells were cultured in the presence or absence of ouabain (30 pM) for 24 h, and filopodia protrusion was captured under fluorescence and phase-contrast modes of a microscope using phalloidin and sulforhodamine B assays, respectively. Fig. 3A shows that H292 cells in the moving state exhibited a substantial number of filopodia protrusions that was dramatically reduced in response to ouabain treatments. These results suggest that the reduction of filopodia in the cells may, at least in part, play a role in the negative regulatory role of ouabain. Sufficient evidence has indicated that filopodia formation in cancer cells involves the Cdc42 protein [26]–[28]. Because the expression level of Cdc42 was shown to be tightly associated with the formation of filopodia, we further investigated the mechanism of ouabain in the regulation of filopodia in these cells by determining the cellular expression of Cdc42. The results indicate that the expression level of Cdc42 dramatically decreased in response to ouabain treatments (Fig. 3B). While an adequate level of this protein was detected in untreated cells, 30 pM ouabain nearly abolished the Cdc42 expression. This information suggests that ouabain may reduce the migratory activity of these cells via Cdc42 attenuation.

### Ouabain Reduces Activation of FAK, Akt, and ERK via a ROS-dependent Mechanism

**Figure 4:**
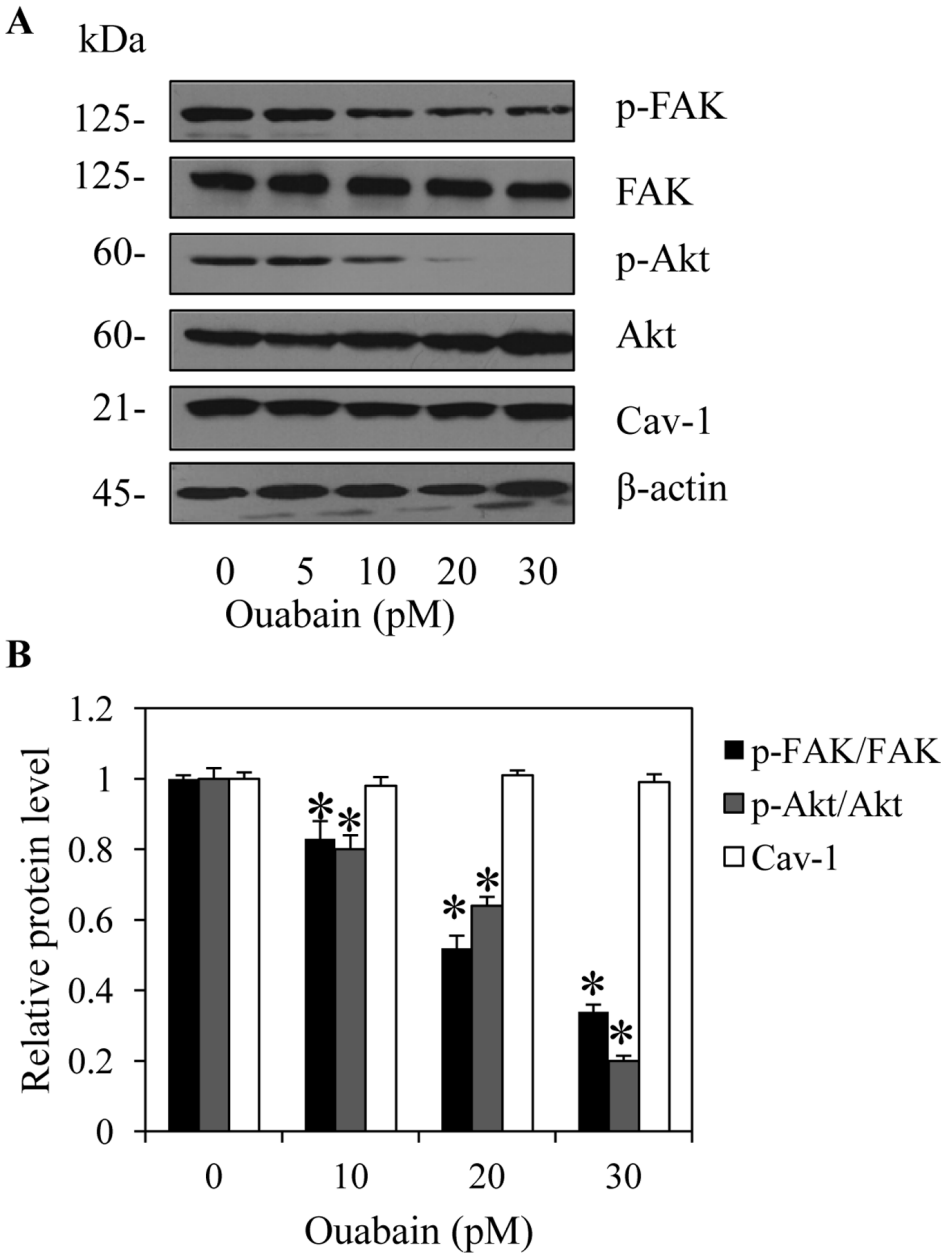
Effect of ouabain on migratory-related proteins. A: H292 cells were treated with ouabain (0–30 pM) for 24 h. The cells were collected and analyzed for focal adhesion kinase (FAK), phospho-FAK (p-FAK, Tyr397), ATP-dependent tyrosine kinase (Akt), phospho-Akt (p-AKT, Ser473), and caveolin-1 (Cav-1) expression by Western blotting. The blots were reprobed with β-actin to confirm equal loading. B: The immunoblot signals were quantified by densitometry, and mean data from independent experiments were normalized to the results. The bars are the means ± SD (n = 4). *P<0.05 vs. untreated control cells.

To elucidate the mechanisms of cancer cell motility inhibition by ouabain, the present study determined the expression and activated levels of proteins involved in cell migration. The cells were treated with non-toxic concentrations of ouabain or left untreated, and Akt, activated Akt (phosphorylated Akt at Ser473) [29], FAK, activated FAK (phosphorylated FAK at Tyr-397) [24] and Cav-1 levels were determined by western blot analysis. Fig. 4 shows that treatment of the cells with ouabain (0–30 pM) substantially down-regulated the expression of activated Akt and activated FAK while preserving their non-activated counterparts. These results suggest that ouabain interfered with the activated level of the proteins and possibly regulated cell migration and invasion by inhibiting Akt and FAK downstream pathways. Recently, we and others have indicated the considerable role of Cav-1 on lung cancer invasive and migratory activities. We further tested whether ouabain attenuates cell motility through decreased Cav-1 levels. Unexpectedly, the level of Cav-1 in response to ouabain treatment was not altered (Fig. 4).

**Figure 5:**
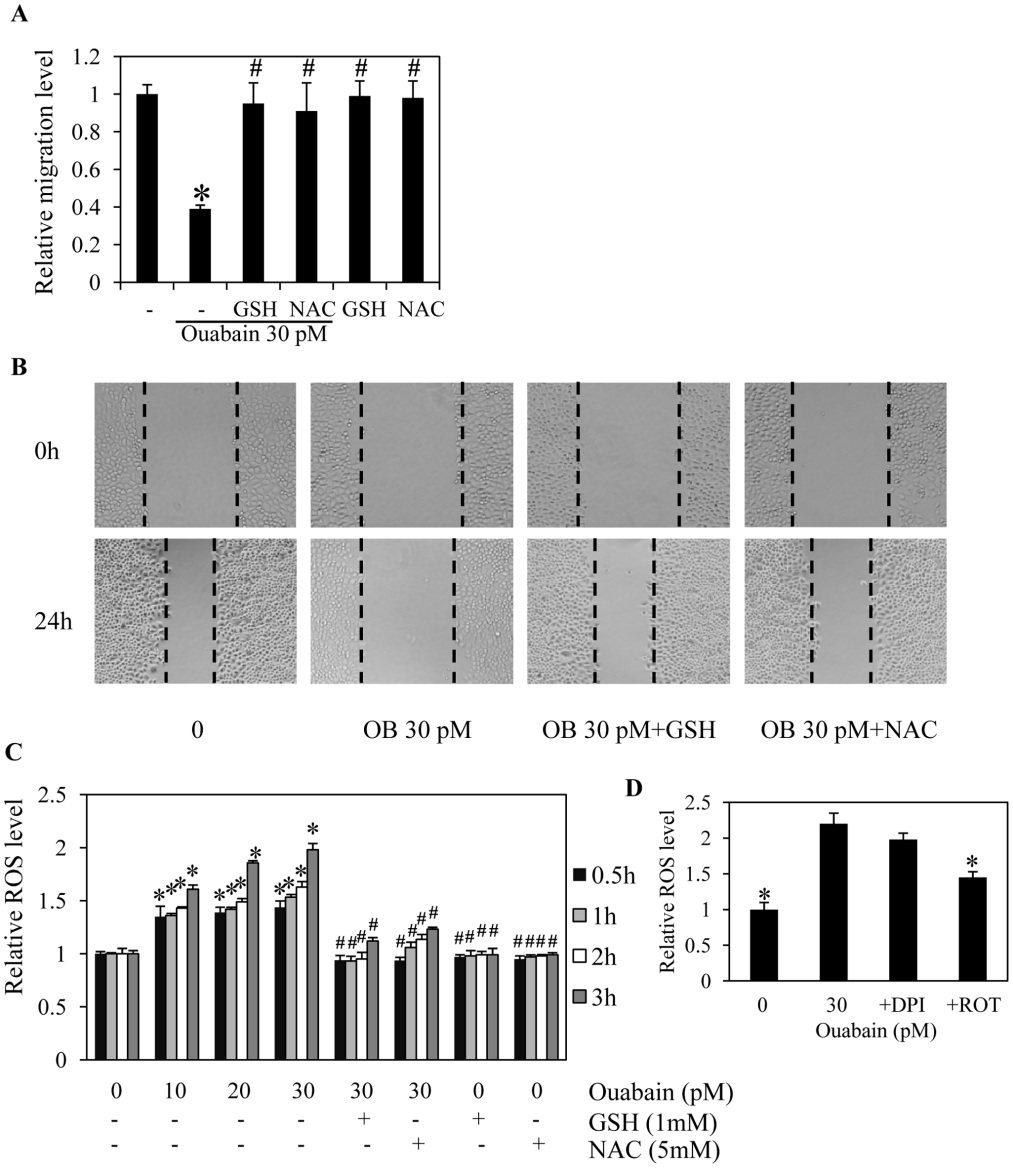
Ouabain inhibits cancer cell migration through reactive oxygen species (ROS) generation. A: Confluent monolayers of H292 cells were wounded by using a 1-mm-wide tip and incubated with antioxidants, 1 mM cell-permeable glutathione (GSH) or 5 mM N-acetyl cysteine (NAC) in the presence or absence of ouabain (0–30 pM) for 24 h. The wound space was analyzed and represented as the migration level relative to the change of the untreated cells. B: Cell migration was captured using a microscope. C: The cells were pretreated with either 1 mM GSH or 5 mM NAC for 30 min in the presence or absence of 30 pM ouabain or ouabain (0–30 pM) alone for 0–3 h. The cells were then incubated with dichlorofluorescein diacetate (DCFH_2_-DA) for 30 min, and the fluorescence intensity was analyzed by a microplate reader. Mean intensity was normalized to untreated control cells and represented as relative ROS levels. Data points are means ± SD (n = 4). *P<0.05 vs. untreated control cells. ^#^ *P*<0.05 vs. 30 pM ouabain-treated cells.

**Figure 6:**
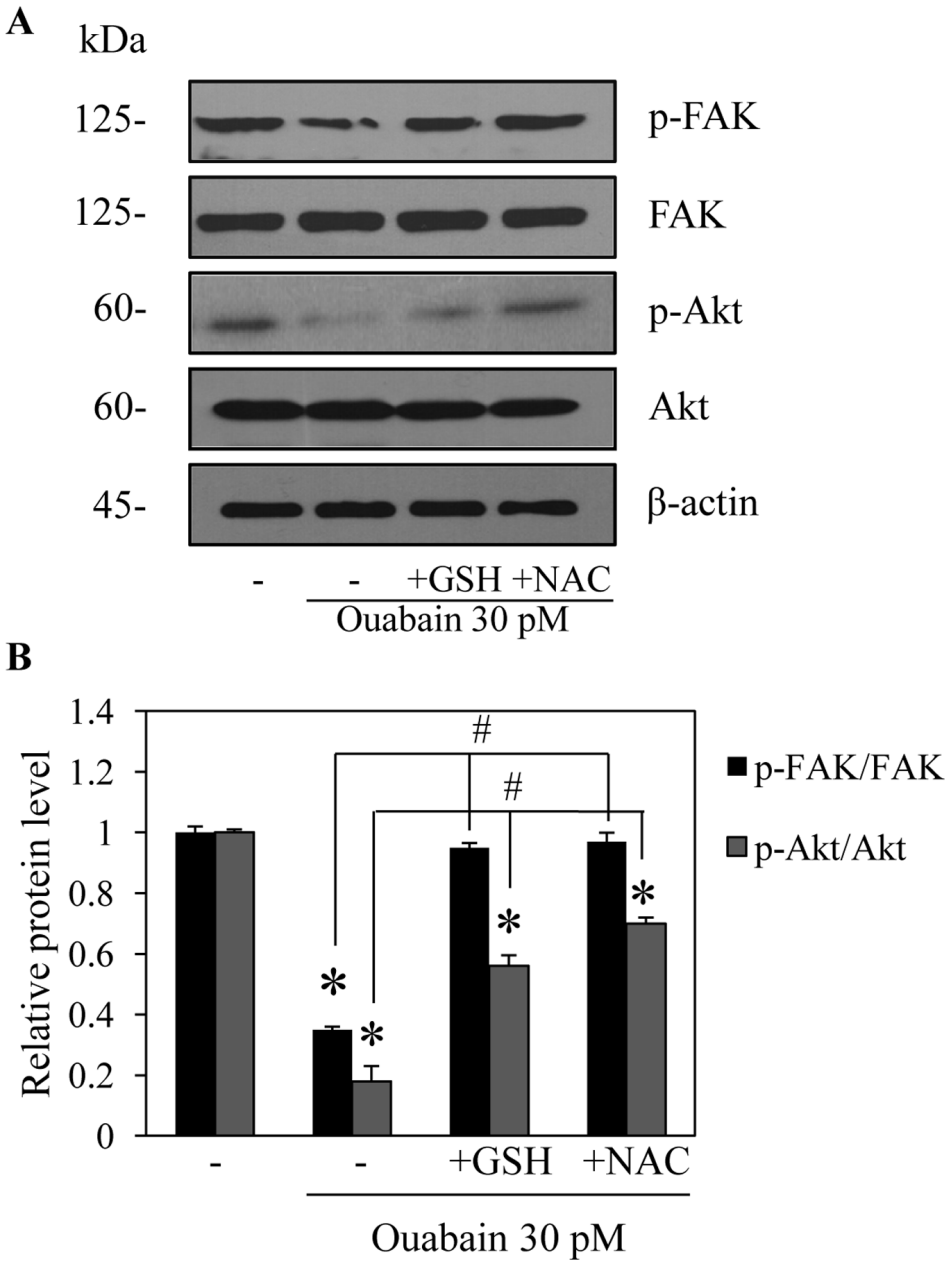
Effect of ROS generated by ouabain on migration-related proteins. A: H292 cells were pretreated with either 1 mM GSH or 5 mM NAC for 30 min in the presence or absence of ouabain (30 pM) for 24 h. The cells were collected and analyzed for focal adhesion kinase (FAK), phospho-FAK (p-FAK, Tyr-397), ATP-dependent tyrosine kinase (Akt) and phospho-Akt (p-AKT, Ser-473) expression by Western blotting. The blots were reprobed with β-actin to confirm equal loading. B: The immunoblot signals were quantified by densitometry, and mean data from independent experiments were normalized to the results. The bars are the means ± SD (n = 4). *P<0.05 vs. untreated control cells.^ #^ *P*<0.05 vs. 30 pM ouabain-treated cells.

We have shown that the migration of cancer cells is tightly associated with the oxidative status of cells. Previous data indicated that the migratory activity of cancer cells was significantly attenuated in response to antioxidant treatments [30]. To clarify the possible regulatory effect of ouabain in this context, the cells were treated with antioxidants in the presence or absence of ouabain and their migratory behavior and cellular ROS levels were assessed. Figs. 5A and 5B show that treatment with the known antioxidants cell-permeable glutathione (GSH) and N-acetylcysteine (NAC) significantly increased migration of the ouabain-treated cells, confirming the role of ROS in the migratory activity of cells that is suppressed by ouabain. In addition, intracellular ROS levels were determined in the cells incubated with ouabain, GSH, and NAC. ROS detected by a specific oxidative probe showed that cellular ROS increased in response to ouabain treatments (10–30 pM), compared with untreated control, whereas neither NAC nor GSH alone had an effect on either cell motility or endogenous ROS levels. Importantly, addition of GSH and NAC in the ouabain-treated cells significantly suppressed the ROS production triggered by ouabain (Fig. 1C). Furthermore, we provided a link between cellular ROS and the activation status of Akt and FAK. The cells were pre-treated with either GSH or NAC in the presence or absence of ouabain and the levels of Akt, activated Akt, FAK and activated FAK were determined as described above. The results indicate that administration of the antioxidants reversed the down-regulating effect of ouabain on activated Akt and activated FAK, whereas the non-phosphorylated forms of both proteins were unaffected (Fig. 6). These findings not only provide evidence of a pro-oxidant effect of ouabain, but they also indicate the possible mechanism of ouabain in inhibiting cell migration via a ROS-dependent mechanism.

### Ouabain Reduces Migration and Invasion of NSCLC H460 Cells

**Figure 7:**
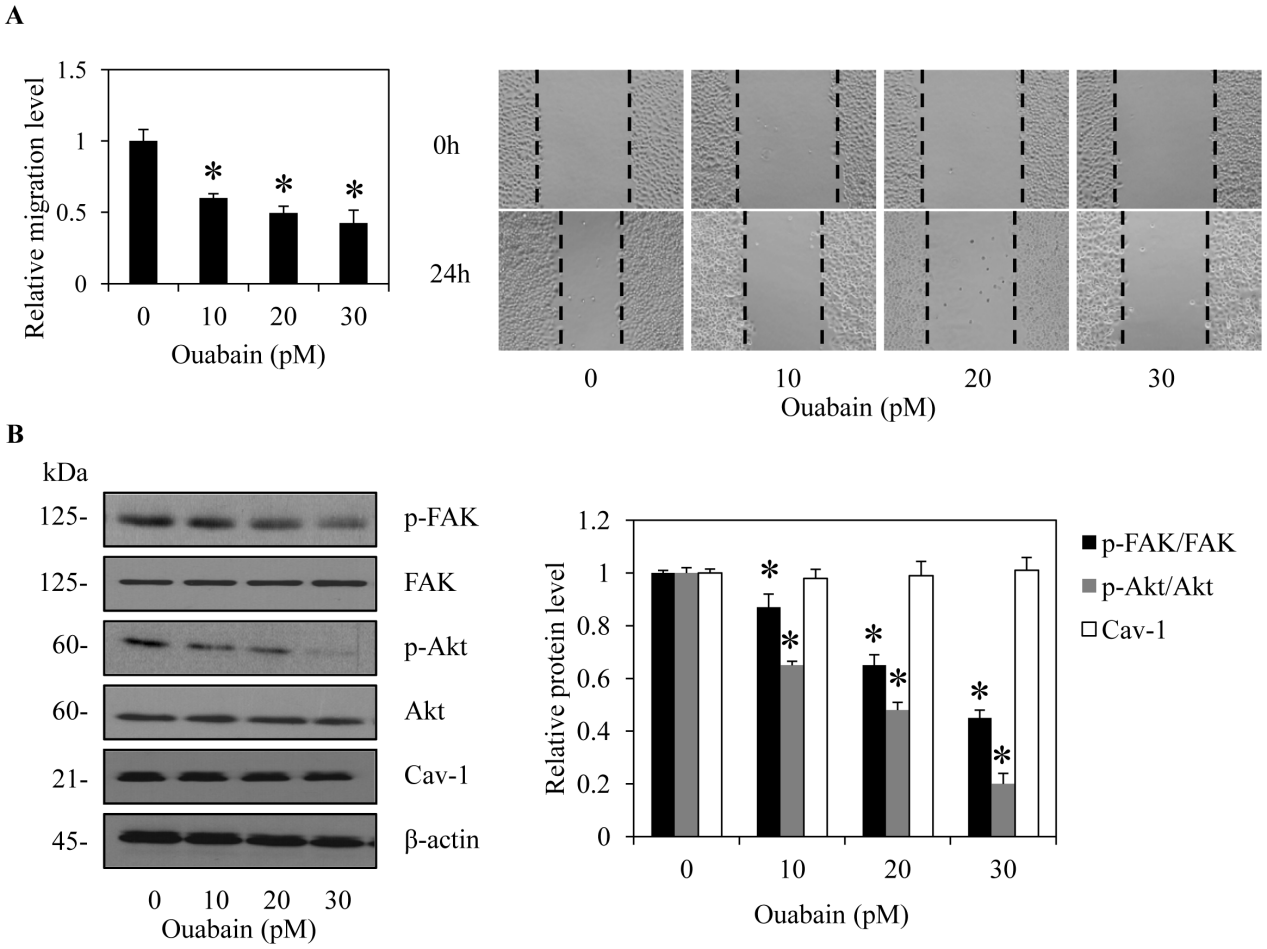
Ouabain inhibition of non-small cell lung cancer (NSCLC) H460 cell migration. A: Confluent monolayers of H460 cells were wounded by using a 1-mm-wide tip and incubated with ouabain (0–30 pM) for 24 h. The wound space was analyzed and represented as the migration level relative to the change of the untreated cells. Data are the means ± SD (n = 4). *P<0.05 vs. untreated control cells. B. Under the same treatment conditions, the cells were collected and analyzed for focal adhesion kinase (FAK), phospho-FAK (p-FAK, Tyr-397), ATP-dependent tyrosine kinase (Akt), phospho-Akt (p-AKT, Ser-473) and caveolin-1 (Cav-1) expression by Western blotting. The blots were reprobed with β-actin to confirm equal loading. The immunoblot signals were quantified by densitometry, and mean data from independent experiments were normalized to the results. The bars are the means ± SD (n = 4). *P<0.05 vs. untreated control cells.

To test whether ouabain was able to inhibit migration in other lung cancer cells, we treated the human non-small cell lung cancer cell line H460 with ouabain (0–30 pM) and subjected the cells to a migration assay. Notably, the MTT results using the H460 cells indicated that treatment with ouabain at 0–30 pM caused no significant effect on the viability of the cells (data not shown). Consistent with the results obtained from the H292 cells, treatment of the H460 cells with ouabain at the indicated concentrations significantly attenuated cell migration in a dose-dependent manner (Fig. 7A). Additionally, the expression of proteins and their activated counterparts were determined. Western blotting results indicated that treatment with ouabain similarly altered activated Akt, activated FAK, and activated ERK, while the non-activated proteins and Cav-1 levels were not affected by the treatment (Fig. 7B).

### Ouabain Suppresses Tumor Growth in 3D Tumor Spheroid Condition and Inhibits Cell detachment

**Figure 8:**
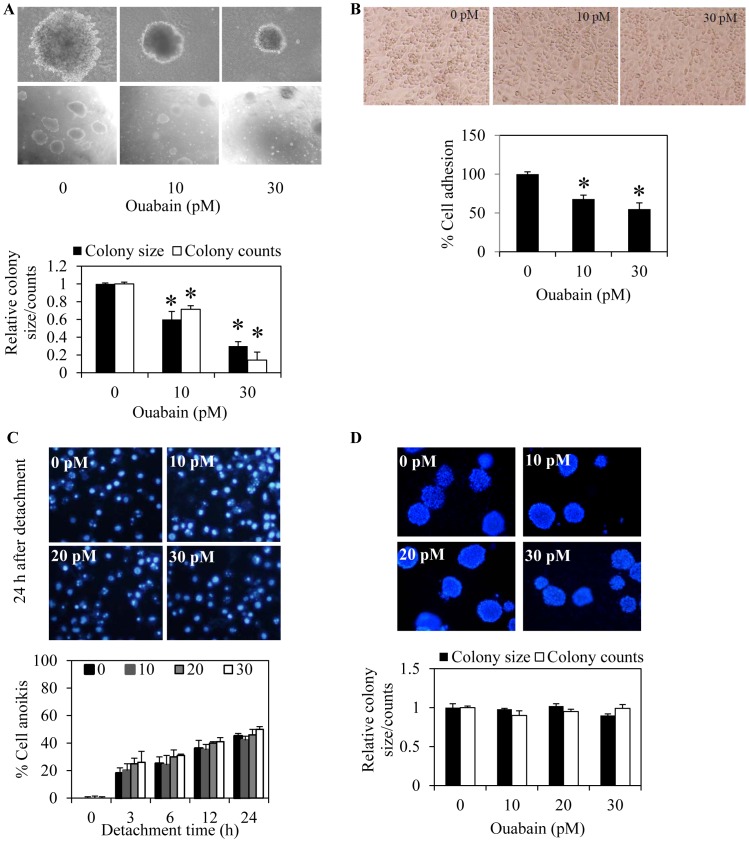
Ouabain suppresses spheroidal tumor growth and cancer cell adhesion to endothelial cells. A: H292 cells were suspended in culture medium containing 4% matrigel and ouabain (0–30 pM) and plated onto matrigel-coated plate. After 10 days, colonies were visualized under microscope with 40× (upper row) and 4× magnifications (lower row), respectively. Value was represented as average diameter and number of colonies in each field relatively to control cells using image analyzer. Data are the means ± SD (n = 4). *P<0.05 vs. untreated control cells. B: H292 were pretreated with various concentration of ouabain (0–30 pM) for 24 h, then, detached H292 cells were subjected to a monolayer culture of HUV-EC-C endothelial cells. The interaction of H292 and HUV-EC-C cells was examined in the presence of IL-1β (10 ng/ml). Phase-contrast microscopic pictures of representative experiments are shown. Data are means ± SD (n = 4), *P<0.05 vs. nontreated control cells. C: H292 cells were detached and treated with ouabain (0–30 pM). Cells were stained with Hoechst for 30 min and visualized under fluorescent microscope. Data are the means ± SD (n = 4). *P<0.05 vs. untreated control cells at each time point. D: H292 cells were mixed with medium containing ouabain (0–30 pM) and subjected to soft agar colony formation assay for 2 weeks as describe under “Material and Methods”. Colonies were observed under fluorescent microscope. Data are the means ± SD (n = 4). *P<0.05 vs. untreated control cells.

Having shown the role of ouabain in cancer cell migration, we next investigated the potential regulation of cancer metastasis by ouabain. The effect of ouabain on key metastasis steps which are anoikis, cell detachment, and growth in 3D tumor spheroid condition were evaluated. Because previous studies have shown that *in vitro* 3D tumorigenesis assay provides proximate *in vivo* cancer condition and the cells grown as a spheroid activate signaling pathways associated with cancer progression and metastasis *in vivo* (16, 30–31), we therefore investigated whether ouabain affects the growth of the lung cancer cells in this manner. Figure 8A shows that ouabain caused a significant decrease in size and number of colonies formed, suggesting its negative regulatory role on cancer cell growth and survival in the tumor spheroid condition. Ouabain was also tested for its activity on cell detachment to endothelial cells. Results indicated that treatment of the cells with ouabain decreased the number of adhere H292 cells on monolayer of HUV-EC-C endothelial cells significantly in a concentration-dependent manner (Fig. 8B). However, ouabain exhibited only minimal effect on the anoikis characteristic of the cells. Cells treated with ouabain at the indicated concentrations showed no significantly alteration in terms of cell survival after detachment in comparison with nontreated cells (Fig. 8C). To confirm, cells were similarly treated with ouabain, subjected to anchorage-independent growth assay, and size and number of the cell colony at day 14 was evaluated. Consistent with the anoikis assay, our results showed that ouabain caused no effect on the growth and survival of the cells in anchorage-independent condition compared with nontreated control (Fig. 8D). These results indicated the potential effect of ouabain as a promising anti-metastasis agent.

## Discussion

While the normal migration of cells is required for several physiological processes, including tissue repair and regeneration and embryonic development, this cell behavior also drives the pathological progression of cancer metastasis [31]. In lung cancer, a high degree of metastasis has been shown to cause high mortality. Therefore, therapeutic means that suppress cancer cell metastasis are considerably important in the lung cancer-related field. A previous study demonstrated the role of ouabain, a human hormone, on the sensitization of lung cancer cells to TRAIL-induced apoptosis [7]. Ouabain levels in human plasma and tissue distribution from different studies seem to be varied in the range of 0.002–0.77 nM [1]–[3]. Herein, we demonstrate that endogenous concentrations of ouabain play a role in inhibiting lung cancer cell migration and invasion.

The migration of cancer cells involves multiple cellular signals, and most of them involve the invasive function of the cells. While the exact mechanisms are still being investigated, several proteins including FAK, Akt and Cav-1 have been shown to control motility in cancer cells. Among several regulatory proteins, tyrosine kinases, such as focal adhesion kinase (FAK), have been shown to play central roles in cell motility by transmitting signals to the downstream cellular machinery via their kinase activity [24]. Phosphorylation of FAK at Tyr-397 is a prerequisite for its activated state, and phosphorylated FAK was shown to be elevated in highly motile and invasive cancer cells [32]. In the present study, we demonstrated that phosphorylated FAK was reduced in ouabain-treated cells. Although FAK has been indicated as a redox-sensitive protein and increased ROS levels from exogenous H_2_O_2_ enhance FAK activation [33], it was also reported that oxidative stress caused inactivation of several protein tyrosine phosphatases including FAK [34]. Treatment with oxidized low density lipoprotein (oxLDL) shows an increase in endogenous ROS levels, while pretreatment with the antioxidant NAC was able to abrogate this effect in the oxLDL-treated group, consequently triggering FAK activation and promoting cell motility [34]. Our observation supports the later concept that, in certain conditions, ROS play a negative role in FAK activation [34].

Even though many pieces of evidence support the function of the mentioned proteins separately and the pathways regulating cancer cell motility are still largely unknown, a substantial number of studies have determined their regulatory impact on each other. Activated FAK was shown to activate Akt and ERK, and these activations play a key role in cell migration [35]. Based on these data, it is possible that ouabain may suppress FAK activation and then cause a reduction in Akt function through its downstream effectors [24], [35].

Recently, Cav-1, a major protein of the plasma membrane domain named caveolae, has been reported to potentiate cancer cells’ ability to resist anoikis [36]–[37] and to migrate and invade [30]. However, results from the present study indicated that ouabain attenuated the migratory activity of H292 and H460 cells via a Cav-1-independent mechanism (Figs. 4 and 7B).

Furthermore, we provided information regarding effects of ouabain on key steps of cancer metastasis including anoikis, detachment, growth in 3D condition, and cancer cell adhesion to endothelial cells. Tumor Growth in 3D was proposed to mimic the pathological condition of the cancer *in vivo* and several studies indicated that the cancer cells grown in a spheroid exhibits signaling pathways and cell behaviors observed in the process of cancer metastasis *in vivo* [38]–[40]. Ouabain was shown to inhibit several key steps of cancer metastasis, namely cell growth in 3D tumorigenesis assay and adhesion to endothelial cells (Fig. 8). However, we found that ouabain has insignificant effect on cancer cell anoikis. These findings have supported the anti-metastasis potentials of ouabain in the physiological concentrations.

Because information regarding endogenous substances that play a significant role in cancer cell behaviors could contribute to the knowledge of the disease, the present study provides important information that may support a better understanding of lung cancer and lead to the development of novel therapies.
